# Lyme arthritis of the pediatric lower extremity in the setting of polyarticular disease

**DOI:** 10.1007/s11832-014-0602-3

**Published:** 2014-07-23

**Authors:** Amiethab Aiyer, William Hennrikus, Jessica Walrath, Brandt Groh, Barbara Ostrov

**Affiliations:** 1 Department of Orthopaedic Surgery, Penn State University/Milton S Hershey Medical Center, 30 Hope Dr, Hershey, PA 17033 USA; 2 Department of Pediatric Rheumatology, Penn State University/Milton S Hershey Medical Center, 30 Hope Dr, Hershey, PA 17033 USA; 3 Penn State College of Medicine, Penn State University/Milton S Hershey Medical Center, 30 Hope Dr, Hershey, PA 17033 USA

**Keywords:** Lyme arthritis, Poly-articular involvement, Clinical algorithm

## Abstract

**Background:**

Lyme arthritis can be readily treated with use of oral antibiotics without any need for surgery. In Lyme-endemic areas, differentiating between Lyme arthritis and septic arthritis can be difficult. Laboratory testing for Lyme disease often results in a delay in diagnosis because many labs batch-test Lyme specimens only two times per week due to lack of equipment or increased expense. Delayed diagnosis can lead to unneeded surgery in cases in which the surgeon indicates the patient for a joint irrigation and debridement (I & D) for possible septic arthritis while waiting for Lyme serology results. The purpose of this study was to develop an algorithm for the treatment of patients with possible Lyme arthritis, with particular attention to poly-articular involvement.

**Methods:**

Thirty-nine patients with poly-articular Lyme arthritis, including ankle involvement, were reviewed retrospectively. Patients were included if the ankle was involved, if they were less than 18 years of age, and had available laboratory information and a serologic diagnosis of Lyme disease.

**Results:**

Only two patients had isolated ankle involvement; of those with poly-articular involvement, 34 patients had ankle/knee involvement. Nine patients presented with pain in the ankle with passive range of motion (PROM) (22 %); two (4.8 %) had refusal to bear weight, and 10 (24 %) had an antalgic gait. All patients had a positive Western blot. Ten patients had a peripheral white blood cell (WBC) count >12,500/mm^3^ , and 16 patients had an erythrocyte sedimentation rate (ESR) >40 mm/h.

**Conclusion:**

Without immediate availability of Lyme serology, the decision to perform surgical drainage of a swollen joint in the setting of possible Lyme arthritis versus septic bacterial arthritis remains a clinical dilemma. Our data suggests that patients presenting with one or fewer Kocher criteria symptoms, poly-articular disease, and minimal pain with PROM have Lyme, rather than septic, arthritis. These patients can be treated with joint aspiration for cultures, appropriate antibiotics for Lyme disease, and careful serial exams while waiting for results of Lyme serology rather than immediate surgical I & D.

## Introduction

Lyme disease is an important consideration in the evaluation of the pediatric patient with a limp. In 60 % of patients who go untreated for Lyme disease, arthritis will be the hallmark feature, presenting several months after the initial tick bite [1]. Lyme disease is recognized as the most common tick-borne disease in the US; per the Centers for Disease Control (CDC), there were approximately 19,000 cases in 2006 [1]. The pediatric orthopaedist, rather than the pediatric rheumatologist, is often the first specialist to see a patient with Lyme arthritis because the patient often presents for an evaluation to rule out septic arthritis. This is of particular importance considering that Lyme arthritis responds to medical management with oral antibiotic administration. Rapid diagnosis of Lyme disease is often delayed, secondary to the delays in obtaining the results of serology testing. Unneeded I & D surgery may be performed in cases in which the patient’s presentation is consistent with septic arthritis or Lyme disease.

On clinical presentation of a patient with Lyme disease, the knee is involved in over 90 % of cases. Other joint involvement is less common. To our knowledge, there is limited description about Lyme arthritis of other joints (including the ankle) in the current literature. Furthermore, there are no reports in which the polyarticular nature of Lyme arthritis is used as a diagnostic criterion. The goal of this study is to review our experience with Lyme arthritis of the ankle in children, particularly in patients with poly-articular involvement, and to develop an algorithm for the treatment of children with possible Lyme arthritis.

## Methods

This study was approved by the Penn State College of Medicine Institutional Review Board. This study, therefore, has been performed in accordance with the ethical standards laid down in the 1964 Declaration of Helsinki and its later amendments.

**Fig. 1:**
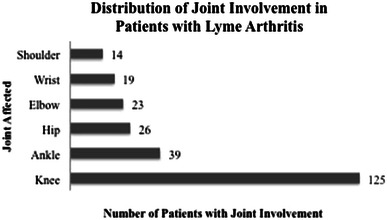
Representative joint involvement in 155 patients with Lyme disease. The knee was most frequently involved, with the ankle second most commonly involved

Patient (pt) clinical records were retrospectively reviewed from 2005 to 2011 for Lyme disease. Our Children’s Hospital is a large tertiary referral center in a Lyme-endemic area. Inclusion criteria included those aged less than or equal to 18, a diagnosis of Lyme (along Centers for Disease Control guidelines, see Table 1), and the availability of laboratory information within our electronic medical system. Exclusion criteria included patients older than age 18 and lack of laboratory information. Information assessed from the clinical record included the following: age, gender, county of residency, oral temperature, joints involved, pain with range of motion (ROM) of affected joint, ability to bear weight on affected joint, whether a joint aspiration was completed, history of a tick bite, history of a rash, recent antibiotic usage, and final treatment. Laboratory information gathered included: erythrocyte sedimentation rate (ESR), white blood cell count (WBC) (with differential), blood cultures, joint cultures (if available), and Lyme titers. A total of 155 patients with Lyme disease were identified. The ankle was found to be the second most commonly involved joint (Fig. 1). For example, 39 patients demonstrated Lyme arthritis of the ankle (25 %). In addition, poly-articular Lyme disease involving the ankle joint was common. One-hundred fifty-three patients (98.7 %) had more than one joint involved, and 37 patients (23.8 %) with poly-articular disease had ankle involvement.

**Table 1:** CDC definition of Lyme Disease [12]

Clinical case definition
Erythema migrans, or
At least one advanced manifestation, as defined below, and laboratory confirmation of infection
Advanced Manifestations (not including CV/CNS)
Musculoskeletal System
Recurrent, brief attacks (lasting weeks or months) of objective joint swelling in one or a few joints, sometimes followed by chronic arthritis in one or a few joints
Manifestations not considered criteria for diagnosis include chronic progressive arthritis, not preceded by brief attacks, and chronic symmetrical polyarthritis
Arthralgia, myalgia, or fibromyalgia syndromes alone are not criteria for musculoskeletal involvement
Laboratory criteria for diagnosis
Positive culture for *B. burgdorferi* from clinical specimen, or
Demonstration of diagnostic levels of IgM and IgG antibodies to the spirochetes in serum
Two-tier testing interpreted using established criteria, where:
Positive IgM is sufficient only when ≤30 days from system onset
Positive IgG is sufficient at any point during illness
Single tier IgG immunoblot seropositivity using established criteria, or
CSF antibody positive for *B. burgdorferi* by enzyme immunoassay (EIA) or Immunofluorescence Assay (IFA), when the titer is higher than it was in serum

## Results

One-hundred and fifty-five patients were found to have Lyme disease. The knee was most commonly affected, and the ankle was the second most commonly affected. The hip, the elbow, the wrist, and the shoulder were less frequently involved (Fig. 1).

Data regarding the initial clinical presentation is listed in Table 2. The 39 patients who presented with ankle involvement had an average age of 9.64 years (range 2–18 years). Only two (2/39, 5.1 %) patients had isolated ankle involvement. Six patients (6/39, 15 %) recalled a tick bite, and 11 (11/39, 28 %) recalled a rash typical of Lyme disease. Nine (9/39, 23 %) patients presented with pain in ankle with range of motion. Pain was graded as minimal, mild, or maximal; pain was found to be maximal unless denoted otherwise. Additionally, two (2/39, 5.1 %) refused to bear weight, and 10 (10/39, 25.6 %) had an antalgic gait. When the combination of pain in other joints and antalgia was further evaluated, 11 (11/39, 28.2 %) patients had pain but no antalgic gait. Six (6/39, 15.3 %) had pain and an antalgic gait, and four (4/39, 10.2 %) had no pain but had an antalgic gait. Only three (3/39, 7.6 %) patients were febrile (>38.5 °C) on presentation. All patients had evidence of swelling of the affected joints.

**Table 2:** Clinical presentation of 39 patients presenting with poly-articular lyme disease with ankle involvement

Pt	Age	M/F	Joints involved	Pain with range of motion	Fever >38.5 °C	Refusal to bear weight
1	17	M	Ankles, knees, neck	No	No	No
2	13	F	L ankle	No	No	No
3	8	F	R ankle, R knee	Yes (R knee)	No	No
4	7	M	Ankles, hips	No	No	No
5	8	F	Ankles, L knee	No	No	No
6	13	M	Ankles, L knee, R wrist	No	No	No
7	5	M	L ankle, L knee, L hip, L knee, L shoulder	No	No	No (antalgic gait)
8	7	F	R ankle, knees, L hip	Yes (R ankle, knees)	No	No
9	17	F	Ankles, wrists	No	No	No
10	14	M	Ankles, knees	Yes (R ankle)	No	No (antalgic gait)
11	12	M	Ankles	No	No	No
12	4	M	L ankle, L knee	Yes (L knee)	No	No
13	13	M	R ankle, L knee, L wrist, neck	Yes (L knee)	No	No (antalgic gait)
14	6	F	Ankles, R hip	Yes (R hip, minimal)	No	Yes
15	12	M	Ankles, knees, back, neck, jaw	Yes (ankles)	No	No
16	18	F	Ankles, knees, elbows, wrists, MCPs	Yes (MCPs, R wrist, 4th and 5th toes)	No	No
17	10	F	Ankle, knees	Yes (L ankle, L knee)	No	No (antalgic gait)
18	9	M	Ankles, knees, wrists, R thumb	No	No	No
19	8	M	L ankle, L knee, L hip	Yes (L ankle, L knee, L hip, mild)	No	No
20	9	F	Ankles, knee, foot, elbows, neck	No	No	No
21	12	F	R ankle, R knee, R shoulder, R elbow, R wrist	Yes (R ankle, R knee, R wrist)	No	No
22	5	F	R ankle, R knee	No	No	No (antalgic gait)
23	5	M	R ankle, R knee, R hip	No	No	No (antalgic gait)
24	10	M	Ankle, shoulder, elbow, wrist, finger	Yes (R ankle)	No	No
25	12	M	L ankle, R knee, R elbow, neck	Yes (L ankle, R knee)	No	No
26	6	M	Ankle, R knee, R foot, elbow, wrist	Yes (R knee)	No	Yes
27	7	F	R ankle, knees, elbows, wrists	Yes (L shoulder, R wrist, R knee)	Yes	No
28	2	F	R ankle, knees, elbows	No	No	No
29	11	F	R ankle, R knee	Yes (R knee, mild)	No	No
30	7	F	L ankle, knees, L hip, L ankle	Yes (L knee)	Yes	No (antalgic gait)
31	9	M	Ankle, L elbow, wrists, neck	No	No	No
32	12	M	R ankle, R knee, L wrist	No	No	No
33	6	M	R ankle, R knee, hips	No	Yes	No (antalgic gait)
34	12	F	L ankle, knees, L midfoot	Yes (L ankle, R knee)	No	No (antalgic gait)
35	7	F	R ankle, R knee, R hip, R shoulder, R wrist, neck	No	No	No
36	14	M	L ankle, L knee, R elbow	Yes (R elbow, L knee)	No	No (antalgic gait)
37	9	F	Ankle, knee, shoulder, wrist	No	No	No
38	9	F	R ankle, R knee, R wrist, R thumb	No	No	No
39	11	F	Ankles, knees, elbows, L 5th finger	No	No	No

Laboratory and treatment data are listed in Table 3. All 39 of the patients had a positive immunoglobulin (IgG) Western blot for Lyme disease. Enzyme-linked immunosorbent assay (ELISA) testing was available for 13 of the 39 patients. Nine of 39 patients had a peripheral WBC count >12,500/mm^3^ (median 10,350/mm^3^, range 3,500/mm^3^–16,300/mm^3^ for the 28 patients with WBC values); 15/39 patients had an ESR >40 mm/h (median 44 mm/h, range 2–90 mm/h for the 31 patients with ESR values), and 7/39 patients had a C-reactive protein (CRP) >4.0 mg/L (median 4.7 mg/L, range 0.51–14 mg/L for the 11 patients with CRP values). All patients were treated with antibiotics. If the treating physician was having a difficult time differentiating between Lyme or septic arthritis, IV antibiotics were started; only 5/39 patients were treated with IV antibiotics. One patient (number 14) underwent a surgical irrigation and debridement procedure while waiting for Lyme serology results to return.

**Table 3:** Lyme laboratory results and treatment of 39 patients presenting with polyarticular lyme disease with ankle involvement

Pt	Western blot (IgG)	ELISA	Antibiotic treatment
1	+	3.25	PO doxycycline
2	+	1.65	PO doxycycline
3	+	None	IV ceftriaxone, amoxicillin
4	+	None	Amoxicillin
5	+	None	Amoxicillin
6	+	None	Doxycycline
7	+	5	Amoxicillin
8	+	None	PO doxycycline
9	+	2.25	Cefuroxime
10	+	None	IV cefuroxime
11	+	None	PO doxycycline
12	+	None	Amoxicillin
13	+	None	PO doxycycline
14	+	None	Amoxicillin
15	+	6	IV ceftriaxone
16	+	None	IV ceftriaxone
17	+	5.8	Amoxicillin
18	+	None	PO doxycycline
19	+	None	IV ceftriaxone
20	+	5	Amoxicillin
21	+	3.1	PO doxycycline
22	+	None	Amoxicillin
23	+	None	Cefuroxamine
24	+	None	PO doxycycline
25	+	None	PO doxycycline
26	+	None	Amoxicillin
27	+	None	Amoxicillin
28	+	None	Cefuroxamine
29	+	4.3	PO doxycycline
30	+	None	Amoxicillin
31	+	None	PO doxycycline
32	+	None	PO doxycycline
33	+	None	Amoxicillin
34	+	8.67	PO doxycycline
35	+	5.6	Amoxicillin
36	+	None	Ceftriaxone
37	+	None	Cefuroxime
38	+	8.2	Amoxicillin
39	+	>5	PO doxycycline

## Discussion

Lyme disease is a common cause of acute arthritis in children in endemic areas. Lyme arthritis responds readily to oral antibiotic management. The difficulty in the evaluation of Lyme arthritis is its clinical similarity to septic arthritis, especially when a patient presents with a single, swollen joint. Although serologic analysis is critical in identifying Lyme arthritis, obtaining these tests often results in a delay in diagnosis and sometimes unneeded surgical I & D. Therefore, the aim of this study was to develop a clinical algorithm to help differentiate Lyme arthritis from septic arthritis, with special attention given to ankle involvement and polyarticular involvement [2, 3].

Kocher identified four factors that could be used to distinguish septic arthritis from transient synovitis of the hip; these included fever >38.5, WBC >12, ESR >40, and an inability to bear weight on the affected leg. Kocher found that if 4/4 criteria were identified, the patient had close to a 99 % chance of having a septic process of the hip [4]. In contrast, if a patient had one out of four criteria, the likelihood of septic process of the hip decreased to 3 %.

Data from the present study, in the context of Kocher’s criteria, revealed that only three patients were found to have a fever >38.5 at the time of presentation. Ten patients (10/39, 25.6 %) had a WBC >12. Although multiple studies have demonstrated that the ESR is elevated in Lyme disease, only 15 patients (15/39, 38.5 %) were found to have values >40 (Table 4). Additionally only 9/39, or 22 %, had pain with passive range of motion. On the other hand, pain with passive range of motion (PROM) is a hallmark of septic arthritis [5].

**Table 4:** Kocher criteria of 39 patients presenting with polyarticular lyme disease with ankle involvement

Pt	Fever >38.5 °C	WBC (/mm3)	Refusal to bear weight	ESR (mm/h)	Kocher criteria
1	No	10	No	8	0/4
2	No	12.8	No	25	¼
3	No	13.7	No	59	2/4
4	No	n/a	No	53	1/4
5	No	11.6	No	90	1/4
6	No	WNL	No	WNL	0/4
7	No	n/a	No	22	0/4
8	No	5	No	17	0/4
9	No	5.6	No	90	1/4
10	No	4.6	No	2	0/4
11	No	n/a	No	n/a	0/4
12	No	11	No	45	1/4
13	No	WNL	No	WNL	0/4
14	No	12.6	Yes	90	3/4
15	No	6.61	No	n/a	0/4
16	No	WNL	No	n/a	0/4
17	No	WNL	No	44	1/4
18	No	6.6	No	15	0/4
19	No	13	No	36	1/4
20	No	7.6	No	12	0/4
21	No	WNL	No	28	0/4
22	No	15.5	No	75	2/4
23	No	n/a	No	n/a	0/4
24	No	n/a	No	n/a	0/4
25	No	9	No	50	1/4
26	No	3.5	Yes	15	1/4
27	Yes	16.3	No	65	3/4
28	No	14.1	No	53	2/4
29	No	10.7	No	50	1/4
30	Yes	13.9	No	81	3/4
31	No	n/a	No	33	0/4
32	No	n/a	No	n/a	0/4
33	Yes	14.6	No	45	3/4
34	No	11.3	No	30	0/4
35	No	13.8	No	54	2/4
36	No	8.3	No	22	0/4
37	No	8	No	4	0/4
38	No	4.6	No	32	0/4
39	No	7.2	No	n/a	0/4

In the present study, we applied the Kocher criteria to patients with ankle and polyarticular involvement. We found that no patients had 4/4 Kocher criteria. Four patients had 3/4 Kocher criteria, and four patients had 2/4 Kocher criteria. Ten patients had 1/4 Kocher criteria, and 21 patients had 0/4 Kocher criteria. Although the Kocher criteria was developed for evaluating the pediatric hip, data from the present study suggest that these criteria are useful for differentiating Lyme from septic arthritis when the ankle and multiple joints are involved. In the current cohort of patients, the lack of Kocher criteria indicated the absence of a bacterial infectious process.

A recent multivariate analysis by Milewski in a Lyme-endemic area indicated that refusal to bear weight is the most predictive factor of septic arthritis [5]. Prior reports in the literature also suggested that refusal to bear weight is an important predictive factor of septic arthritis [6, 7]. This is consistent with the results of the present study. Furthermore, we concur with Culp that refusal to bear weight is rare with Lyme disease; in our study, only two patients (2/39, 5.1 %) with Lyme disease of the ankle refused to bear weight. However, these two patients did not have pain with passive range of motion, which indicated Lyme arthritis rather than septic arthritis. In the present study, the presence of pain with passive motion was seen in six patients and an antalgic gait was seen in 11 patients [8].

An algorithm to differentiate Lyme disease from septic arthritis may minimize the number of patients who undergo surgical debridement when the diagnosis is not clear. For example, in a 2011 study by Milewski, 40/123 (~24 %) cases of Lyme arthritis underwent operative debridement for presumed septic arthritis [5]. In our study, one patient (number 14) underwent surgical debridement, who was later found to have positive Lyme titers. This patient was subsequently managed with oral antibiotics. The low operative rate (2.5 %) in this study highlights the importance of the need to differentiate between Lyme and septic arthritis.

Williams reported that about 2/3 of patients with Lyme disease present with multiple joint involvement (an average of 2.4 joints affected) [9]. In addition, Williams reported that the ankle was the second most commonly affected joint, after the knee. We concur with Williams that the ankle was the second most commonly involved and report an even higher rate of polyarticular involvement of 94 %.

Comparative studies between Lyme disease and septic arthritis usually focus on a single joint involvement and often apply the Kocher criteria to monoarticular evaluation. In contrast, in the current study, we applied Kocher’s criteria to polyarticular Lyme disease, with emphasis on cases involving the ankle. The rate of isolated ankle involvement is uncommon. Our data indicate that only two (2/39) had isolated ankle involvement. Our data suggest that knee/ankle involvement was the most common combination of joints and was seen in 56 % of cases. The results of this study strongly suggest that this finding of polyarticular involvement indicates Lyme arthritis rather than septic arthritis. The sensitivity of polyarticular involvement related to Lyme disease was 97.4 %.

There are several salient points that our data affords for analysis. First, in the context of Kocher’s criteria, patients with Lyme disease rarely had an elevated temperature, an elevated ESR, an elevated WBC, or difficulty bearing weight. The lack of Kocher criteria was very suggestive of Lyme arthritis. Historical clues are unreliable, since only 17 % of our patients reported a tick bite and 31 % noted a rash. This lack of historical clues is consistent with previous reports in the literature [1].

We suggest the following algorithm to help differentiate between Lyme and septic arthritis. Patients with two or fewer Kocher criteria, polyarticular disease, an ability to bear weight, and minimal pain with passive range of motion are more likely to have Lyme disease and should be treated with appropriate antibiotics and careful follow-up while waiting for Lyme serology results. Patients with three or more Kocher criteria, monoarticular involvement, inability to bear weight, and pain with passive range of motion of the joint are more likely to have septic arthritis and should be treated with surgical I and D, cultures, and appropriate IV antibiotics, also while waiting for Lyme serology results.

Limitations include the retrospective design of the study from a single institution and the lack of a control group. This study is the first to evaluate the polyarticular nature of Lyme disease as a tool to help differentiate Lyme arthritis from septic arthritis. Although patients 14, 27, 30, and 33 demonstrated 3/4 Kocher criteria, the polyarticular nature of their presentation convinced the treating surgeon to manage the patients without surgery in three of four of these cases.

An additional limitation of this algorithm is its inability to differentiate Lyme arthritis from juvenile idiopathic arthritis, which can have a similar clinical presentation [10]. The mainstay of differentiating between Lyme and JIA is serologic testing. This is of particular importance in Lyme-endemic regions [10, 11].

## Conclusion

Lyme arthritis is treated with oral antibiotics alone, whereas septic arthritis warrants an irrigation and debridement procedure and IV antibiotics. Similarities in clinical presentation implicate the need for developing an algorithm to differentiate between these pathologies. Unnecessary trips to the operating room may be prevented if the diagnosis is made in a timely fashion. Lyme serology titers are often run by the lab only twice per week, secondary to lack of equipment and expense. Subsequently, the results are not timely when deciding if surgical irrigation/debridement is needed. The key points of this study are as follows:

1. The ankle is the second most commonly involved joint in Lyme disease.
2. In a Lyme-endemic area, polyarticular involvement indicates Lyme arthritis rather than septic arthritis.
3. The Kocher criteria are helpful to differentiate Lyme arthritis from a septic joint.
4. The development of a rapid Lyme test that can be performed in a few hours is needed.
